# Draft Genome Sequence of a Novel Methylobacterium brachiatum Strain Isolated from Human Skin

**DOI:** 10.1128/MRA.01093-20

**Published:** 2020-12-10

**Authors:** Geert Cremers, Angelique Kenyon, Niels Gubbels, Matthijs Jansen, Theo A. van Alen, Tom Berben, Huub J. M. Op den Camp

**Affiliations:** a Department of Microbiology, IWWR, Radboud University, Nijmegen, The Netherlands; University of Southern California

## Abstract

Methylobacterium brachiatum MBRA is an aerobic alphaproteobacterium isolated from the human skin on methanol-containing minimal medium. The genome was sequenced using Illumina and Nanopore technology, and the genome was assembled using Unicycler. *M. brachiatum* MBRA possesses two *xoxF* genes, one gene pair, *mxaF* and *mxaI*, and a complete serine pathway.

## Abstract (precis)

Methylobacterium brachiatum MBRA is an aerobic alphaproteobacterium isolated from the human skin on methanol-containing minimal medium. The genome was sequenced using Illumina and Nanopore technology, and the genome was assembled using Unicycler. *M. brachiatum* MBRA possesses two *xoxF* genes, one *mxaF/mxaI*, and a complete serine pathway.

## ANNOUNCEMENT

Methanol is a by-product of plant (1) and healthy human metabolism (2). Facultative methylotrophic bacteria from the genera *Methylobacterium* and *Methylorubrum* have been isolated from the plant phyllosphere (1), soil (3), and the human body (4–6). Methylobacterium brachiatum was first isolated from a water sample from a food processing plant in Japan (7). Here, we present a draft genome of *M. brachiatum* MBRA, which we isolated from human skin as part of a laboratory course.

Strain MBRA was isolated by placing a human thumb (Nijmegen area, The Netherlands) on solid mineral salt medium (MSM) containing (per liter) 15 g agar, 1.5 g KH_2_PO_4_, 7.9 g Na_2_HPO_4_·2H_2_O, 0.8 g NH_4_Cl, 0.1 g MgSO_4_·7H_2_O, 0.5% methanol, 1 ml 1,000× SL10 trace element solution (8), 0.1 ml 10 mM CeCl_3_ solution, and 0.02 ml 50 mM nitrilotriacetic acid (NTA) solution (medium pH set at 6.8). After growing at room temperature for 1 week, a single colony was picked and restreaked onto an MSM plate. After 5 d at 25°C, biomass was scraped from the plate, and DNA was isolated using a DNeasy PowerSoil kit (Qiagen, Venlo, The Netherlands). Long-read sequencing was performed with the MinION R9 flow cell (FLO-MIN106; Nanopore, Oxford, UK), according to the manufacturer’s protocol, using unsheared DNA (no size selection) and NEBNext formalin-fixed, paraffin-embedded (FFPE) repair mix (M6630), a ligation sequencing kit (SQK-LSK109), the NEBNext end repair/dA-tailing module (E7546), a flow cell priming kit (EXP-FLP001), NEB Blunt/TA ligase master mix (M0367), the NEBNext quick ligation module E6056, and barcode kit EXPNBD104. MiSeq sequencing (Illumina, San Diego, CA, USA) was performed with the Nextera XT kit according to the manufacturer’s protocol. All DNA was measured using a Qubit fluorometer (Thermo Fisher Scientific, Waltham, MA, USA). The quality of the 2,329,486 paired-end Illumina reads (average, 253 bp) was checked using CLC Genomics Workbench v12 (Qiagen Aarhus A/S, Denmark). Read error correction was performed using SPAdes v3.13 (9) in the Unicycler v0.4.4 (10) pipeline, so no trimming was completed prior to assembly. Nanopore reads were base called and demultiplexed using Guppy v3.0.7 (minimum length, 3,000 bp), leaving 129,787 reads with an average length of 8,234 bp for assembly.

Assembly using reads from both methods in Unicycler (10) resulted in 25 contigs with an *N*_50_ value of 1,578,407 bp. Eight contigs were manually binned by GC content (GC content, ∼69%) and Illumina read-based coverage (coverage, ∼50×) (Table 1). Unicycler also identified five circular high-copy-number small contigs, most likely plasmids. The completeness of the genome was checked using CheckM v1.0.11, indicating 97.2% completeness at the *Rhizobiales* level and 0% contamination (11). Average nucleotide identity (ANI) analysis by JSpeciesWS v3.6.1 (12) placed the genome at 97.9% similarity to *M. brachiatum*. The genome was annotated using Prokka v1.12 (13) and manually curated. Default parameters for all programs were used except where otherwise noted.

**TABLE 1:** Characteristics of the Methylobacterium brachiatum MBRA genome sequence and plasmids

Characteristic	Data for:					
	Genome	Plasmid 1	Plasmid 2	Plasmid 3	Plasmid 4	Plasmid 5
Size (bp)	6,459,278	53,490	48,251	45,734	24,909	16,067
DNA GC content (%)	69.4	61.4	68.0	64.9	67.0	56.1
No. of contigs	8	1	1	1	1	1
*N*_50_ (bp)	1,573,792	53,490	48,135	45,734	24,910	16,067
Circular	No	Yes	Yes	Yes	Yes	Yes
Total no. of genes	6,236	57	60	64	25	23
Protein coding density (%)	84.5	85.6	88.9	85.7	84.5	84.9
No. of rRNA genes	15	0	0	0	0	0
No. of tRNA genes	67	0	0	0	0	1
Coverage (×)^*a*^	50	107	117	149	194	296

a Based on MiSeq reads.

The genome sequence of strain MBRA reflects its facultative methylotrophic lifestyle. For growth on methanol, the genome encodes two lanthanide-dependent XoxF-type methanol dehydrogenases and a calcium-dependent MxaFI type (14), together with the recently discovered *lanM* gene (15) and the full serine pathway for carbon assimilation (1). Strain MBRA harbors a glycolysis pathway and a near-complete tricarboxylic acid (TCA) cycle. In addition, the genes for both dissimilatory and assimilatory nitrate reduction were present.

### Data availability.

This whole-genome shotgun sequencing project has been deposited in ENA under accession number PRJEB35543. The assembled genome (13 contigs) is deposited under accession numbers CACTHX010000001 through CACTHX010000013. The versions described in this paper are the first versions. The raw reads are available under SRA accession numbers ERX3765083 and ERX3765085.
